# Aging reduces calreticulin expression and alters spontaneous calcium signals in astrocytic endfeet of the mouse dorsolateral striatum

**DOI:** 10.1038/s41514-023-00102-8

**Published:** 2023-03-31

**Authors:** Sara M. Zarate, Taylor E. Huntington, Pooneh Bagher, Rahul Srinivasan

**Affiliations:** 1 Department of Neuroscience & Experimental Therapeutics, Texas A&M University School of Medicine, 8447 Riverside Pkwy, Bryan, TX 77807 USA; 2 Texas A&M Institute for Neuroscience (TAMIN), Texas A&M University, College Station, TX 77843 USA; 3 Department of Cellular and Integrative Physiology, University of Nebraska Medical Center, Omaha, NE 68198 USA

**Keywords:** Parkinson's disease, Ageing

## Abstract

Aging-related impairment of the blood brain barrier (BBB) and neurovascular unit (NVU) increases the risk for neurodegeneration. Among various cells that participate in BBB and NVU function, calcium signals in astrocytic endfeet are crucial for maintaining BBB and NVU integrity. To assess if aging is associated with altered calcium signals within astrocytic endfeet of the dorsolateral striatum (DLS), we expressed GCaMP6f in DLS astrocytes of young (3–4 months), middle-aged (12–15 months) and aging (20–30 months) mice. Compared to endfeet in young mice, DLS endfeet in aging mice demonstrated decreased calreticulin expression, and alterations to both spontaneous membrane-associated and mitochondrial calcium signals. While young mice required both extracellular and endoplasmic reticulum calcium sources for endfoot signals, middle-aged and aging mice showed heavy dependence on endoplasmic reticulum calcium. Thus, astrocytic endfeet show significant changes in calcium buffering and sources throughout the lifespan, which is important for understanding mechanisms by which aging impairs the BBB and NVU.

## Introduction

Advanced age is the single greatest risk factor for neurodegenerative conditions such as Parkinson’s disease (PD), Alzheimer’s disease (AD)^1^, stroke^2,3^, and dementia^4^. A large body of evidence strongly suggests that aging-related impairment of the blood brain barrier (BBB) and neurovascular unit (NVU) potentiates and possibly triggers neurodegenerative processes in the brain^5–12^. Among the multiple brain regions prone to neurodegeneration, the striatum is particularly vulnerable to neurovascular dysfunction and neurodegeneration because of its rich vascular supply^13^. This notion is supported by reports showing a higher percentage of lacunar infarcts in the striatum compared to other brain regions^14^, increased propensity for accumulating toxic striatal protein aggregates^15,16^, and an increase in striatal BBB permeability during PD, AD, and stroke^17–19^. Furthermore, aging-related changes in the striatal BBB and NVU can lead to parkinsonian symptoms^20^, enlarged perivascular spaces^21^, and PD due to dopaminergic neuron loss^22^. Although these studies indicate that striatal BBB and NVU dysfunction are important contributing factors for various forms of neurodegeneration, little is known about the changes to BBB and NVU function that occur across the lifespan.

The BBB and NVU are comprised of distinct cellular elements, viz., neurons, endothelial cells, pericytes and astrocytes, each of which plays important and specific roles in neurovascular function^23–26^. Among these cell types, astrocytes are unique in that they simultaneously contact neurons and vasculature, thus enabling these cells to alter vascular structure and function in response to brain activity^27–30^. Astrocytes perform this vital function via spontaneous Ca^2+^ signals within endfeet that completely ensheathe capillaries^31,32^. Indeed, Ca^2+^ signals in astrocytic endfeet can alter vasoconstriction and dilation^32,33^, mediate vascular repair after injury^34^, regulate brain volume^35^, alter aging-related cognitive behavior via intracellular IP3R2-specific Ca^2+^ signals^36^, as well as maintain NVU coupling^37^. Thus, at a functional level, astrocytic endfoot Ca^2+^ signals govern critical aspects of BBB integrity and NVU function. It follows that aging-related changes in striatal astrocytic endfoot Ca^2+^ signals likely contribute to BBB and NVU dysfunction during neurodegeneration. Based on the strong body of evidence pointing to striatal vascular dysfunction in various aging-related neurodegenerative diseases as well as a central role for astrocytic endfoot Ca^2+^ signals in maintaining BBB integrity and NVU function, we asked if aging is associated with alterations in spontaneous astrocytic endfoot Ca^2+^ events within the dorsolateral striatum (DLS) of acutely extracted live brain slices from young, middle-aged, and aging mice.

We show that when compared to young mice, DLS brain slices of aging mice display dramatic alterations in the kinetics, dynamics, and sources of spontaneous membrane-associated Ca^2+^ events, as well as changes in Ca^2+^ influx into astrocytic endfoot mitochondria. These aging-related alterations in endfoot Ca^2+^ events are associated with significant reductions in the expression of a major endoplasmic reticulum (ER) Ca^2+^ buffering protein, calreticulin (CALR) and astrocyte endfoot mitochondrial mass. Our findings have important implications for understanding how an aging-related reduction in CALR expression can alter astrocytic endfoot Ca^2+^ signals, eventually resulting in vascular dysfunction during aging and aging-related neurodegeneration.

## Results

### Aging alters multiple parameters of spontaneous membrane-associated astrocytic endfoot Ca^2+^ events in the DLS

**Fig. 1:**
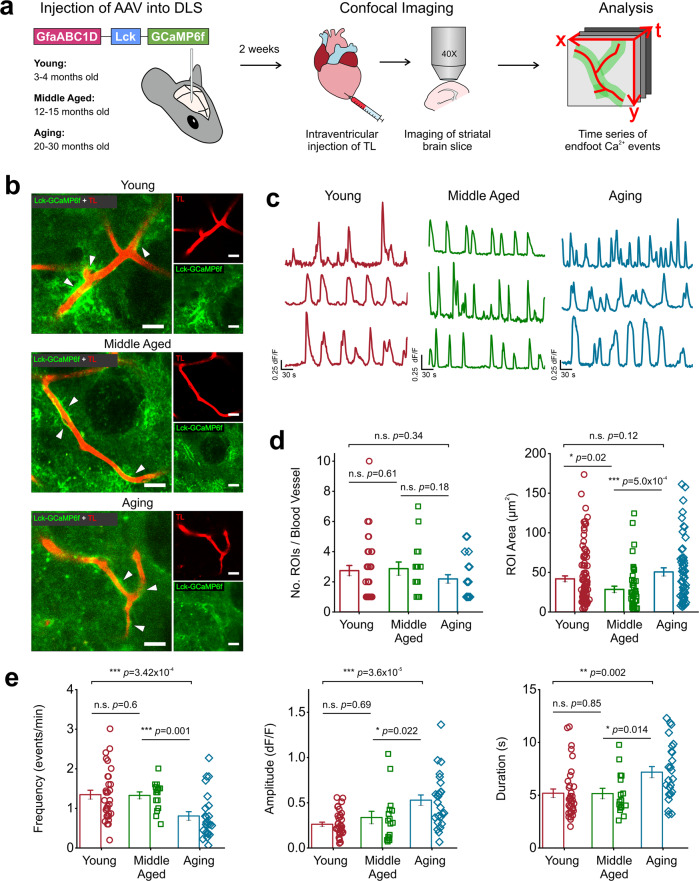
Aging alters spontaneous Ca^2+^ event kinetics. **a** AAV-GfaABC1D-Lck-GCaMP6f was injected into the DLS of young, middle-aged, and aging mice. Two weeks later, intraventricular injection of tomato lectin (TL) was performed, and striatal brain sections were collected for recording and measuring endfoot Ca^2+^ events in the DLS. **b** Representative time stacks of young, middle-aged, and aging astrocyte endfeet expressing lck-GCaMP6f (green) immediately adjacent to TL labeled blood vessels (red) in the DLS, scale bar = 15 μm. White arrows point to areas where endfoot Ca^2+^ events initiated. **c** Example traces of endfoot Ca^2+^ events recorded from young, middle-aged, and aging mice. **d** Comparisons of the number (left) and area (right) of ROIs generated in young, middle-aged, and aging mice. **e** ROIs were averaged across individual blood vessels to compare spontaneous Ca^2+^ event frequency (*left*), amplitude (*middle*), and duration (*right*). For young mice: *n* = 96 ROIs and 36 blood vessels from 7 mice, middle-aged mice: *n* = 46 ROIs and 16 blood vessels from 4 mice, and for aging mice: *n* = 57 ROIs and 26 blood vessels from 7 mice. Error bars are S.E.M and all *p* values are based on Mann-Whitney tests.

We sought to determine whether spontaneous endfoot Ca^2+^ signals in the DLS were altered across the lifespan. To measure spontaneous membrane-associated astrocytic endfoot Ca^2+^ events in the DLS, young, middle-aged, and aging mice were stereotaxically injected with an AAV expressing membrane localized GCaMP6f (Lck-GCaMP6f), driven by an astrocyte-specific GfaABC1D promoter (AAV2/5-GfaABC1D-Lck-GCaMP6f). Intraventricular administration of red fluorescent tomato lectin (TL) into the mouse heart prior to live brain slicing enabled visualization of blood vessels (BV) in the DLS (Fig. 1a) and Lck-GCaMP6f was used to visualize astrocytic endfoot Ca^2+^ events (Fig. 1b). Confocal imaging of live striatal brain slices co-labeled with TL in BVs and Lck-GCaMP6f in astrocytic endfeet revealed that young, middle-aged, and aging mice exhibited robust spontaneous endfoot Ca^2+^ events in ROIs immediately adjacent to TL labeled BVs in DLS brain slices (Fig. 1b and c; Supplemental movies 1–3).

No differences were detected for the three age groups in the number of endfoot Ca^2+^ ROIs (2.74 ± 0.34 ROIs per BV in young, 2.9 ± 0.44 ROIs per BV in middle-aged, and 2.2 ± 0.28 ROIs per BV in aging mice) (Fig. 1d). Conversely, middle-aged endfoot Ca^2+^ ROIs were 31% and 43% smaller than young or aging ROIs, respectively (ROI area = 41.9 ± 3.7 µm^2^ in young, 28.54 ± 4.1 µm^2^ in middle-aged, and 50.6 ± 5.15 µm^2^ in aging mice) (Fig. 1d). Next, we measured spontaneous kinetics (frequency, amplitude, and duration) of DLS astrocyte endfoot Ca^2+^ events in the three age groups. Young and middle-aged mice demonstrated an average endfoot Ca^2+^ event frequency of 1.3 events/min, which was 60% faster than the recorded 0.81 event/min in aging mice (Fig. 1e). Aging mice also showed a 73% increase in Ca^2+^ event amplitude and a 38% increase in event duration compared to both young and middle-aged mice (Fig. 1e). Together these data suggest that aging astrocyte endfeet have a limited capacity to initiate Ca^2+^ fluxes across cellular compartments, regulate the amount of Ca^2+^ fluxing, and reuptake Ca^2+^ once it has been mobilized.

**Fig. 2:**
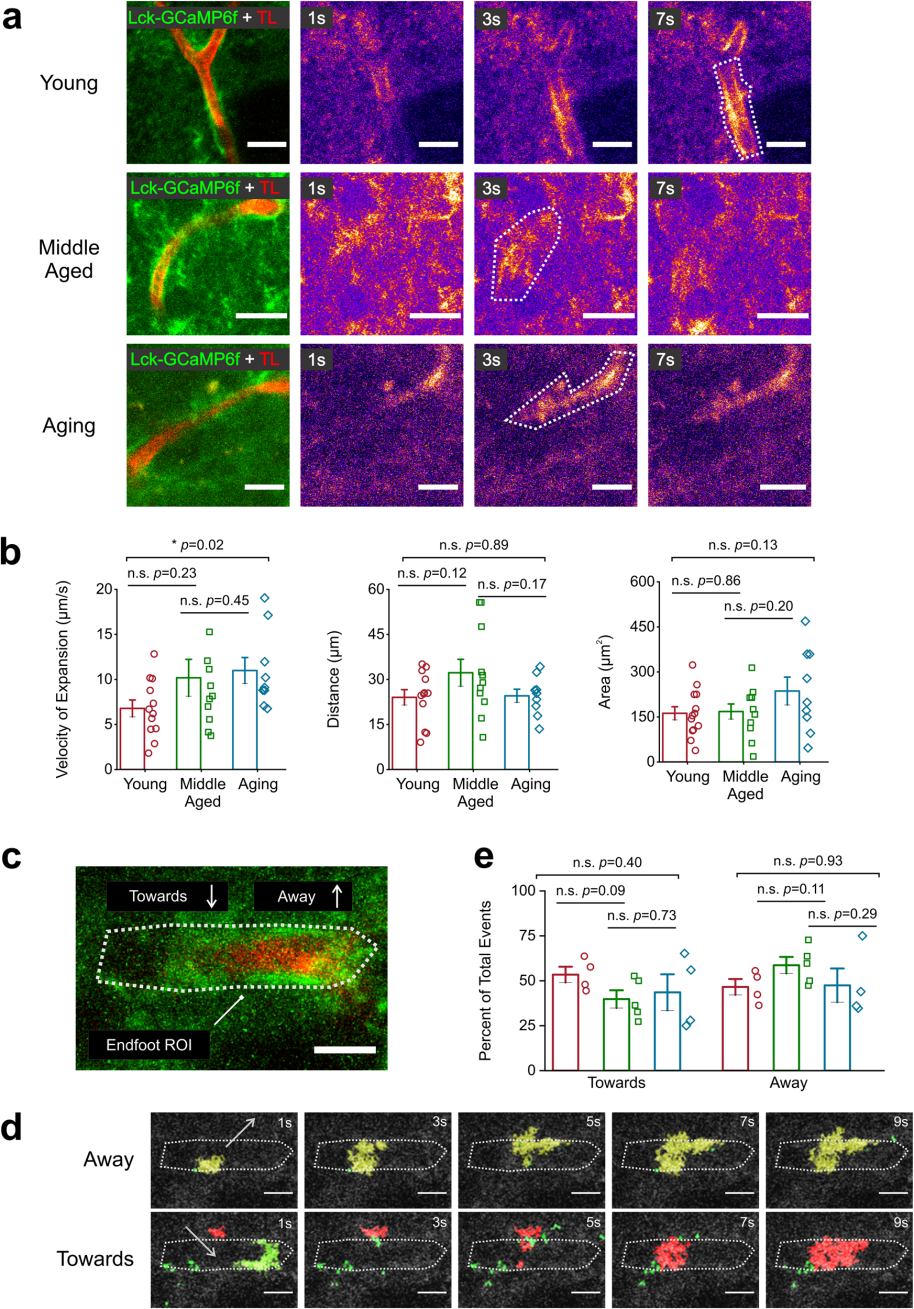
Aging increases the velocity of expanding Ca^2+^ waves in astrocyte endfeet. **a** Representative t-stacks of an expanding endfoot Ca^2+^ wave in the DLS of young (*top*), middle-aged (*middle*), and aging (*bottom*) mice is shown. Panels to the right of t-stacks show representative pseudo-colored time lapse images of endfoot Ca^2+^ waves. The white dotted lines in time-lapse images outline the maximum area attained by expanding endfoot Ca^2+^ waves, scale bar = 15 μm. **b** Bar graphs showing the velocity (*left*), distance traveled (*middle*), and area (*right*) of endfoot Ca^2+^ waves. **c** For analysis using AQuA, ROIs were manually drawn to demarcate the area where events would be analyzed in relation to the BV (white dotted line), scale bar = 10 μm. **d** Example time lapses of events moving away (*top*) or towards (*bottom*) the endfoot ROI, scale bar = 10 μm. (**e**) Bar graph comparing the percentage of expanding Ca^2+^ waves detected by AQuA that either expanded towards or away from the BV. For **a**, **b**, young mice: *n* = 13 ROIs and 5 blood vessels from 3 mice, for middle-aged mice: *n* = 11 ROIs and 4 blood vessels from 3 mice, and for aging mice: *n* = 9 ROIs and 4 blood vessels from 3 mice. For the velocity panel the *p* value is based on a Mann-Whitney test and the distance and area panel *p* values are based on two sample t-tests. For **d**, young mice: n = 4 ROIs and 4 blood vessels from 3 mice, for middle-aged mice: *n* = 5 ROIs and 4 blood vessels from 3 mice, and for aging mice: *n* = 4 ROIs and 4 blood vessels from 3 mice. For comparisons of events moving towards or away from the BV, two-sample *t* tests were used to determine *p* values. All error bars are S.E.M.

In line with previous reports, endfoot Ca^2+^ events in all mice occurred as either static or expanding waves adjacent to TL BVs^38,39^ (Fig. 2a; Supplemental movies 1–3). For these expanding Ca^2+^ waves, the expansion velocity, distance traveled, area covered, and movement towards or away from the BV were analyzed. Compared to young mice, endfoot Ca^2+^ events in both middle-aged and aging mice showed a significant increase in expansion velocity (10.98 ± 1.45 µm/s in aging, 10.18 ± 2.05 µm/s in middle-aged, and 6.8 ± 0.93 µm/s in young mice) but no differences in the distance traveled or area covered (Fig. 2b). We next used AQuA to measure whether expanding Ca^2+^ events were moving towards or away from the BV (Fig. 2c, d; Supplemental movie 4). We found no change in the percentage of events moving towards or away from the BV across all age groups (Fig. 2e). Thus, these data indicate that aging-related changes to astrocyte endfoot expanding Ca^2+^ events are restricted to the velocity by which the Ca^2+^ events propagate along striatal BVs.

Altogether, these data demonstrate widespread age-induced limitations in Ca^2+^ event initiation, buffering, and propagation that likely begin in middle-aged mice and become more pronounced in advanced age.

### Aging alters the dependence of astrocytic endfoot signals on extracellular Ca^2+^ versus ER Ca^2+^ stores

Since Ca^2+^ signals depend on both extracellular and intracellular Ca^2+^ stores, we utilized young, middle aged, and aging mice to determine if there were differences in the contribution of extracellular or intracellular Ca^2+^ sources that feed endfoot Ca^2+^ signals across the lifespan.

**Fig. 3:**
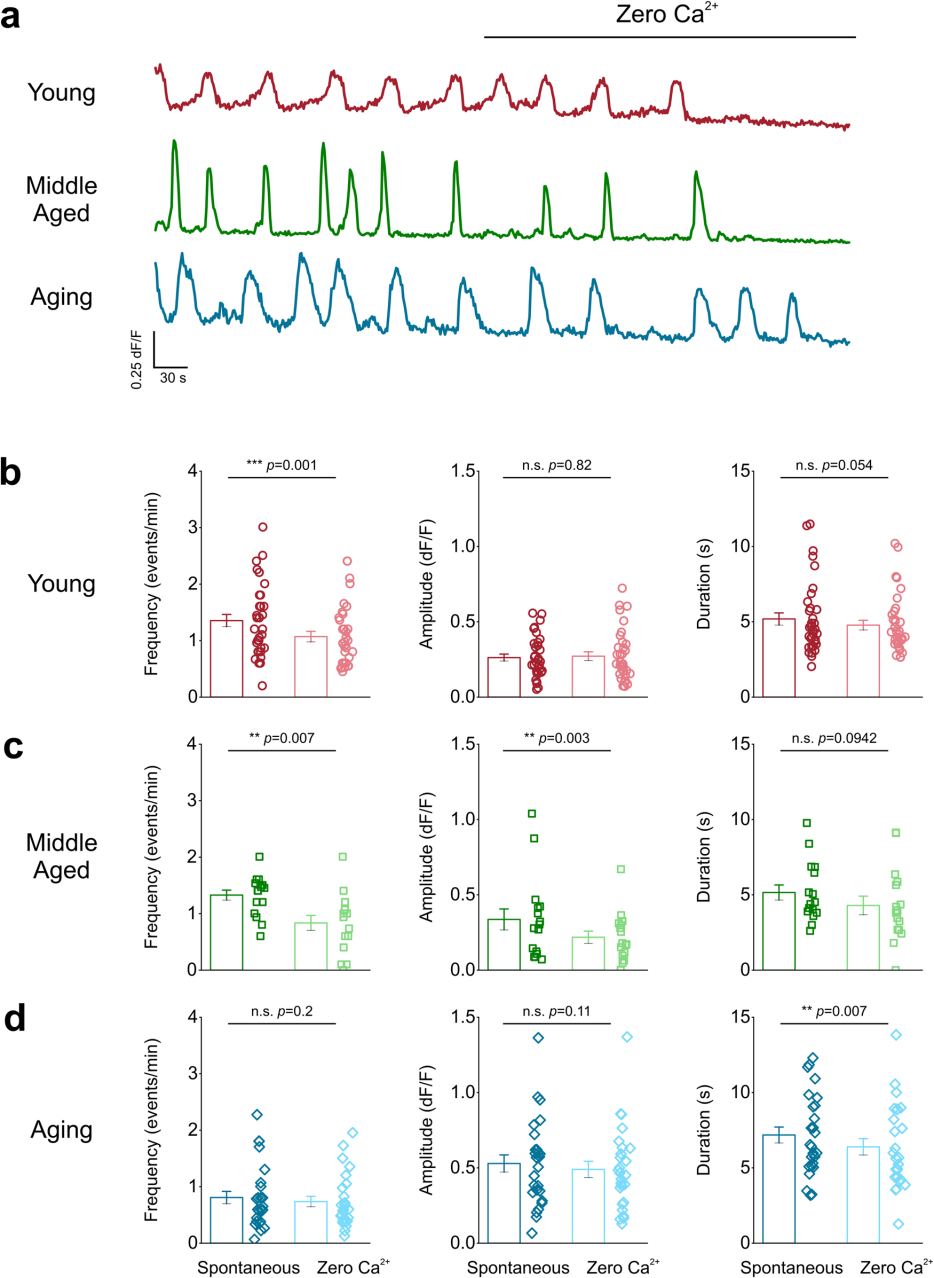
Astrocyte endfeet in aging mice do not rely on extracellular Ca^2+^ to generate spontaneous Ca^2+^ events. **a** Representative endfoot Ca^2+^ event traces from the DLS of young (*top*), middle-aged (*middle*), and aging (*bottom*) mice after bath application of zero Ca^2+^ aCSF. ROIs were averaged across individual blood vessels to compare Ca^2+^ event frequency (*left*), amplitude (*middle*), and duration (*right*) for young (**b**), middle-aged (**c**) and aging (**d**) mice after bath application of zero Ca^2+^ aCSF. For young mice: *n* = 96 ROIs and 36 blood vessels from 7 mice, middle-aged mice: *n* = 46 ROIs and 16 blood vessels from 4 mice, and for aging mice: *n* = 57 ROIs and 26 blood vessels from 7 mice. Error bars are S.E.M. All *p* values for young frequency, amplitude, and duration are based on Wilcoxon signed ranked tests. For middle-aged mice, the frequency and duration panel *p* values are based on paired sample *t* tests and the amplitude panel *p* value is based on a Wilcoxon signed rank test. Frequency and amplitude *p* values for aging mice were derived from Wilcoxon signed rank test and the duration *p* value is based on a paired sample t-test.

Extracellular and intracellular Ca^2+^ stores were sequentially depleted and astrocyte endfoot Ca^2+^ events were recorded. In young mice, depletion of extracellular Ca^2+^ with zero Ca^2+^ aCSF (Fig. 3a; Supplemental movie 1) caused a significant 21% decrease in Ca^2+^ event frequency with no effect on the amplitude or duration of the events (Fig. 3b). Middle-aged mice showed a significant decrease in event frequency and amplitude after depletion of extracellular Ca^2+^ but no change in duration (Fig. 3c; Supplemental movie 2). For aging mice, zero Ca^2+^ aCSF did not affect Ca^2+^ event frequency or amplitude with only a modest decrease in event duration (Fig. 3d; Supplemental movie 3).

**Fig. 4:**
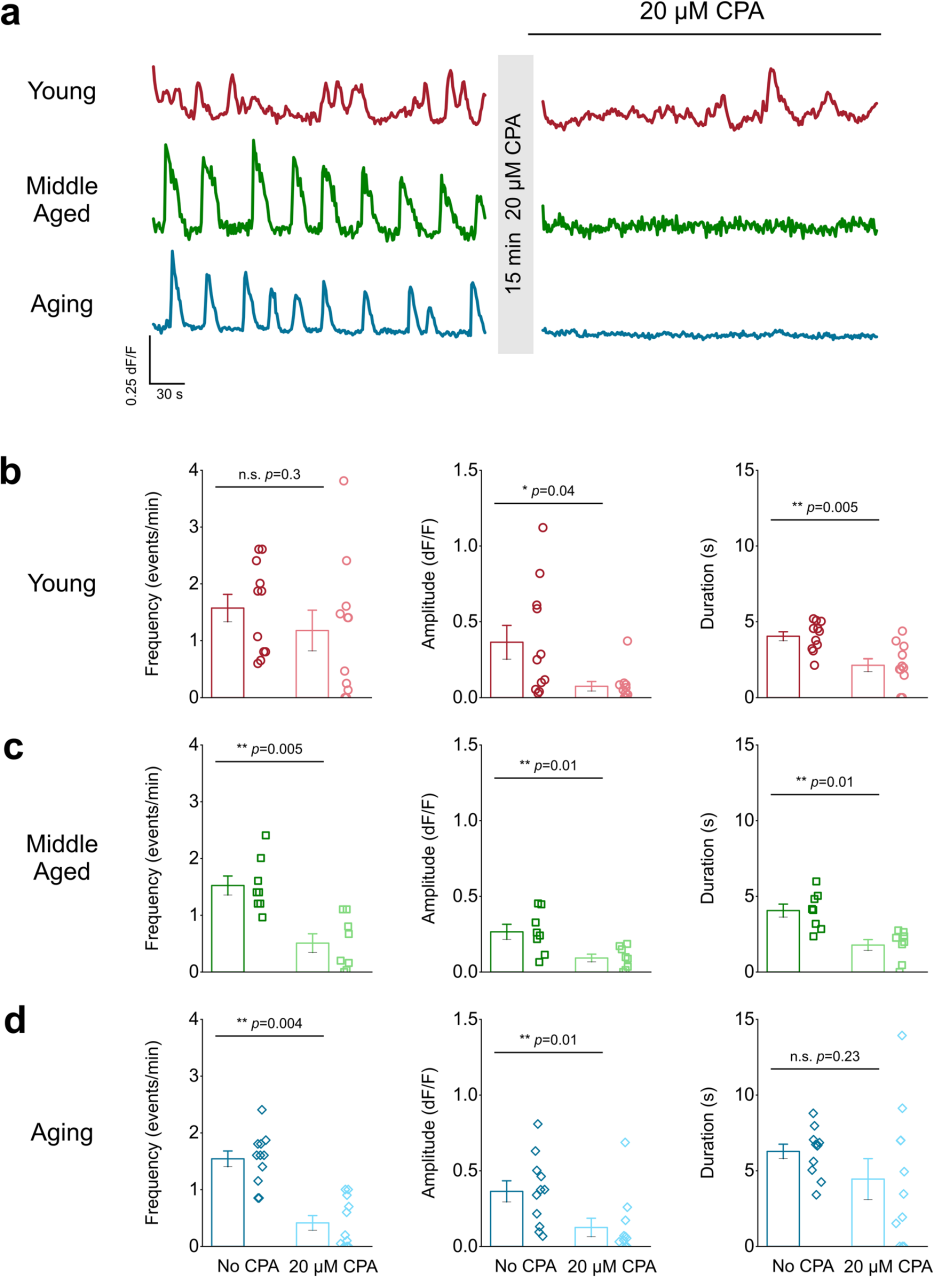
Aging astrocyte endfeet rely exclusively on intracellular ER Ca^2+^ stores to generate spontaneous Ca^2+^ events. **a** Representative endfoot Ca^2+^ event traces from the DLS of young (*top*), middle-aged (*middle*), and aging (*bottom*) mice after 15 min incubation with 20 µM CPA. ROIs were averaged across individual blood vessels to compare Ca^2+^ event frequency (*left*), amplitude (*middle*), and duration (*right*) for young (**b**), middle-aged (**c**) and aging (**d**) mice after 15 min incubation with 20 µM CPA. For young mice: *n* = 21 ROIs and 11 blood vessels from 5 mice, middle-aged mice: *n* = 22 ROIs and 8 blood vessels from 4 mice, and aging mice: *n* = 24 ROIs and 11 blood vessels from 6 mice. Error bars are S.E.M. For young mice frequency and duration *p* values are based on two sample t-tests and the young amplitude *p* value is based on a Wilcoxon signed ranked test. All *p* values for middle-aged frequency, amplitude, and duration are based on paired sample t-tests. Frequency and amplitude *p* values for aging mice were derived from Wilcoxon signed rank test and the duration *p* value is based on a paired-sample t-test.

ER Ca^2+^ stores were depleted with 20 µM CPA, a Ca^2+^-ATPase SERCA pump inhibitor, for 15 min (Fig. 4a). Ca^2+^ event frequency in young mice was not significantly reduced after incubation with 20 µM CPA (Fig. 4b; Supplemental movie 5); however, young mice showed an 81% decrease in event amplitude and a 47% decrease in event duration (Fig. 4b). Conversely, both middle-aged and aging mice showed significant 67% and 74%, respective decreases in endfoot Ca^2+^ events following exposure to CPA (Fig. 4c, d; Supplemental movies 6 and 7). Middle-aged mice exhibited a 67% decrease in event amplitude and a 56% decrease in event duration (Fig. 4c). Aging mice also demonstrated a 64% decrease in event amplitude but no change in the duration of Ca^2+^ events that persisted after CPA application (Fig. 4d).

These data show that in young and middle aged mice, DLS astrocytic endfeet rely on both extracellular and intracellular Ca^2+^ stores for generating and maintaining spontaneous Ca^2+^ events, but a shift to near exclusive reliance on intracellular ER Ca^2+^ stores occurs during advanced aging.

### Aging mice demonstrate a significant reduction in the expression of CALR

**Fig. 5:**
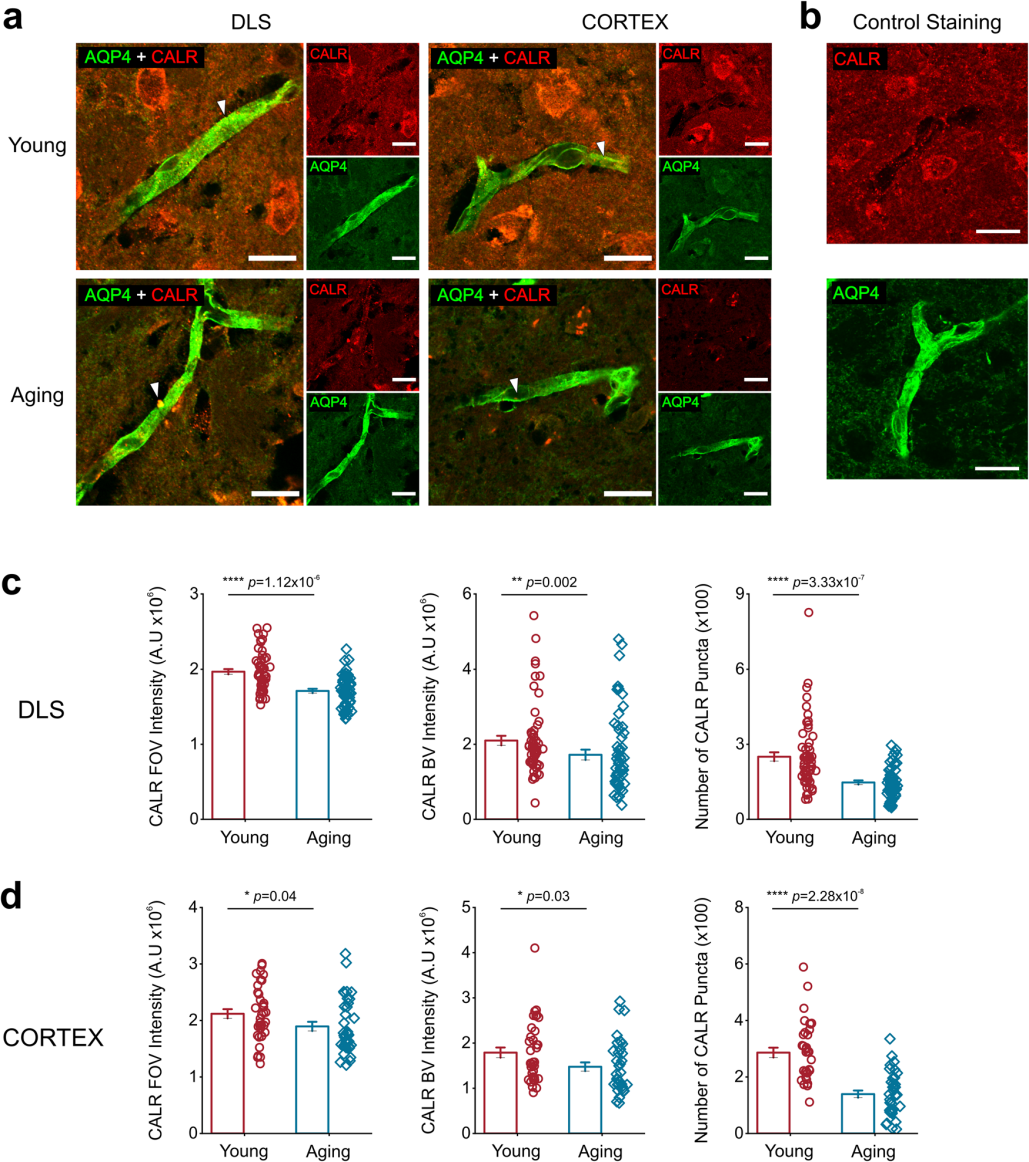
Aging reduces CALR expression levels in astrocyte endfeet and in the neuropil of the DLS and adjacent cortex. **a** Representative single plane confocal images from the DLS or cortex of young (*top*) and aging (*bottom*) mice co-labeled with CALR (red) and AQP4 (green), scale bar = 15 μm. The white arrows point to individual CALR puncta that coincide with AQP4 positive blood vessel staining. **b** Single plane confocal images from young mice stained with either CALR or AQP4 alone, scale bar = 15 μm. Bar graphs comparing neuropil CALR intensity (*left*), CALR intensity within AQP4 positive BVs (*middle left*), the number of CALR puncta in AQP4 positive BVs (*middle right*) in the DLS (**c**) or cortex (**d**). For CALR in the DLS, young and aging mice *n* = 54 blood vessels from 9 striatal sections and 3 mice per age group. For the cortex, young and aging mice n = 36 blood vessels from 9 striatal sections and 3 mice per age group. Error bars are S.E.M, all *p* values are based on Mann-Whitney tests.

Based on our findings that aging alters ER maintained spontaneous endfoot Ca^2+^ events (Figs 1, 2, 3, and 4), we hypothesized that aging could alter expression of the major ER-localized Ca^2+^ buffering protein calreticulin (CALR)^40^ To assess this possibility, young and aging striatal brain sections were co-immunostained for CALR and the astrocyte endfoot marker, aquaporin 4 (AQP4). In both young and aging mice, astrocytic AQP4 labeling clearly outlined BVs, while CALR staining appeared as punctate structures throughout AQP4 stained endfeet and into the surrounding neuropil (Fig. 5a). To rule out cross reactivity between CALR and AQP4 primary and secondary antibodies, a separate set of sections were stained with CALR or AQP4 only. The control staining was identical to co-stained sections suggesting that the antibodies were not co-reactive (Fig. 5b). When compared to young mice, aging mice showed a 13% reduction in CALR throughout the neuropil (Fig. 5c). In addition, CALR intensity was reduced by 18% in aging AQP4 positive BVs and the number of CALR puncta was decreased from 250.23 ± 17.61 in young mice to 147.28 ± 8.23 in aging mice (Fig. 5c).

We next sought to determine if the observed decrease in CALR intensity and puncta was specific to the DLS; thus, we quantified CALR in the cortex just adjacent to the DLS (Fig. 5a). Aging mice showed a similar reduction in cortical CALR intensity throughout the neuropil and corresponding AQP4 BV ROIs as seen in the DLS (Fig. 5d). However, the number of CALR puncta in each BV quantified was reduced by 52% from 286.3 ± 17.48 in young mice to 139.36 ± 12.73 in aging mice as compared to the 40% reduction observed in the aging DLS (Fig. 5d).

Together, these data show that aging causes a significant, widespread reduction in expression of the major Ca^2+^ buffer, CALR in the DLS and cortex.

### Aging increases the frequency of spontaneous Ca^2+^ influx into endfoot mitochondria

**Fig. 6:**
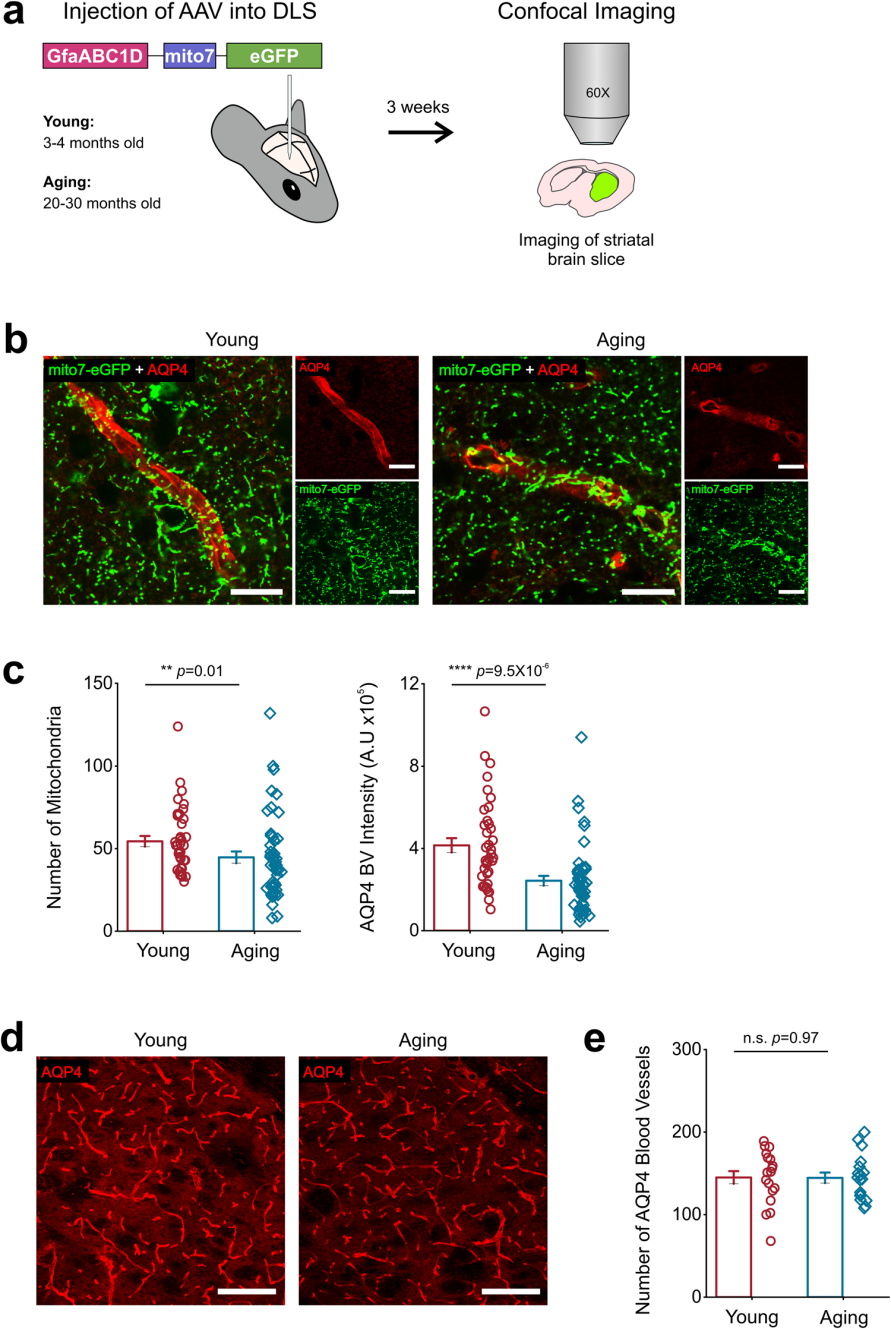
Aging reduces the number of mitochondria in astrocyte endfeet and AQP4 expression in the DLS. **a** AAV-GfaABC1D-mito7-eGFP was injected into the DLS of young and aging mice. 3 weeks later, striatal sections were imaged to obtain single plane images of mitochondria in AQP4 labeled BVs. **b** Representative single plane confocal images from the DLS of young (*left*) and aging (*right*) mice expressing AAV-GfaABC1D-mito7-eGFP (green) and stained with AQP4 (red), scale bar = 15 μm. **c** Bar graph comparing the number of mitochondria in AQP4 labeled BVs (*left*) and AQP4 intensity (*right*) in young and aging mice. **d** Representative z-stacks of blood vessels labeled with AQP4 in the DLS of young (*left*) and aging (*right*) mice, scale bar = 150 μm. **e** Bar graph comparing the number of AQP4 labeled blood vessels in young and aging mice. For mitochondria counting and AQP4 intensity, young mice *n* = 38 blood vessels from 9 striatal sections and 3 mice and aging mice: *n* = 49 blood vessels from 9 striatal sections and 3 mice per age group. Error bars are S.E.M, *p* values are based on Mann-Whitney tests. For the number of AQP4 blood vessels, *n* = 6 DLS FOV from 3 mice each for young and aging mice. Error bars are S.E.M. and the *p* value is based on a two-sample t-test.

We have previously shown that the ER serves as a major source for Ca^2+^ influx into astrocytic mitochondria in the DLS^41^. Thus, we rationalized that aging induced changes to endfoot Ca^2+^ dynamics and CALR expression would likely alter endfoot mitochondria number and function. We injected the DLS of young and aging mice with AAV5-GfaABC1D-mito7-eGFP (Fig. 6a) and stained striatal sections with AQP4 to quantify changes in the number of endfoot mitochondria (Fig. 6b). When compared to young mice, the number of mitochondria in AQP4 positive BVs (Fig. 6b) was reduced by 18%; from 54.45 ± 3.2 in young mice to 44.73 ± 3.56 in aging mice (Fig. 6c). Interestingly, we also observed a significant 80% decrease in AQP4 BV intensity in aging mice as compared to young mice (Fig. 6c) but no change in the total number of vessels present in the DLS (Fig. 6d, e).

**Fig. 7:**
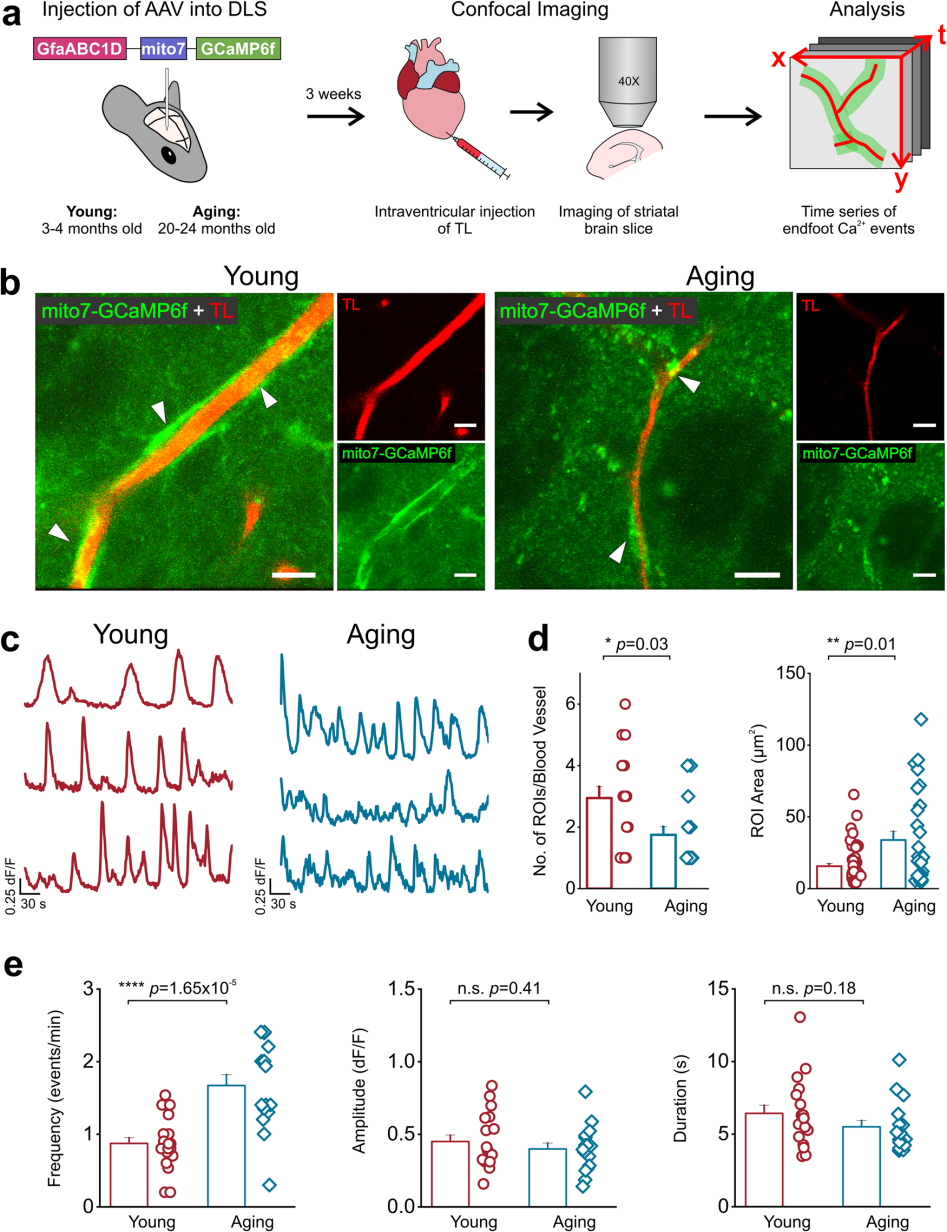
Aging increases the frequency of endfoot mitochondrial Ca^2+^ events. **a** AAV-GfaABC1D-mito7-GCaMP6f was injected into the DLS of young and aging mice. 3 weeks later, intraventricular injection of tomato lectin (TL) was performed, and striatal brain sections were collected for recording and measuring Ca^2+^ influx events in endfoot mitochondria within the DLS. **b** Representative t-stacks of mitochondrial Ca^2+^ influx events detected by mito7-GCaMP6f (green) in young and aging astrocyte endfeet immediately adjacent to TL labeled blood vessels (red) in the DLS, scale bar = 15 μm. White arrows point to areas where mitochondrial endfoot Ca^2+^ events initiated. **c** Representative endfoot mitochondrial Ca^2+^ influx event traces from young (red) and aging (blue) mice. **d** Bar graphs showing the number (*left*) and area (*right*) of endfoot mitochondrial Ca^2+^ event ROIs in young and aging mice. **e** ROIs were averaged across individual blood vessels to compare endfoot mitochondria Ca^2+^ event frequency (*left*), amplitude (*middle*), and duration (*right*) in young and aging mice. For young mice, *n* = 53 ROIs and 18 blood vessels from 3 mice. For aging mice, *n* = 28 ROIs and 16 blood vessels from 5 mice. Error bars are S.E.M and all *p* values in (**d**) are based on Mann-Whitney test. In (**e**), frequency and amplitude *p* values are based on two-sample t-tests and the duration panel *p* value is based on a Mann-Whitney test.

Because the number of DLS astrocyte endfoot mitochondria was reduced in aging mice, we next sought to quantify endfoot mitochondria Ca^2+^ events in the DLS. Thus, young and aging mice were stereotaxically injected with a previously described construct, AAV2/5-GfaABC1D-mito7-GCaMP6f^41^, and confocal imaging was performed in live striatal brain slices from young and aging mice with TL as a marker for BVs (Fig. 7a, b). Endfoot mitochondria in young and aging mice displayed robust spontaneous Ca^2+^ influx events (Fig. 7b and c; Supplemental movies 8 and 9). However, when compared to young mice, aging mice showed a 41% decrease in the number of endfoot mitochondrial Ca^2+^ event ROIs and a very large ~ 116% increase in the size of ROIs (Fig. 7d).

There were also significant aging-related changes in the kinetics of mitochondrial Ca^2+^ influx events. When compared to young mice, aging mice demonstrated a dramatic 92% increase in the frequency of endfoot mitochondrial Ca^2+^ influx (Fig. 7e). For both young and aging mice, there were no significant alterations in the amplitude or duration of spontaneous mitochondrial Ca^2+^ influx events (Fig. 7e). Thus, aging-related increases in ER Ca^2+^ specifically caused dramatic increases in the area and frequency of Ca^2+^ influx in DLS endfoot mitochondria.

### Ca^2+^ influx into endfoot mitochondria of the DLS depends on ER Ca^2+^ stores

**Fig. 8:**
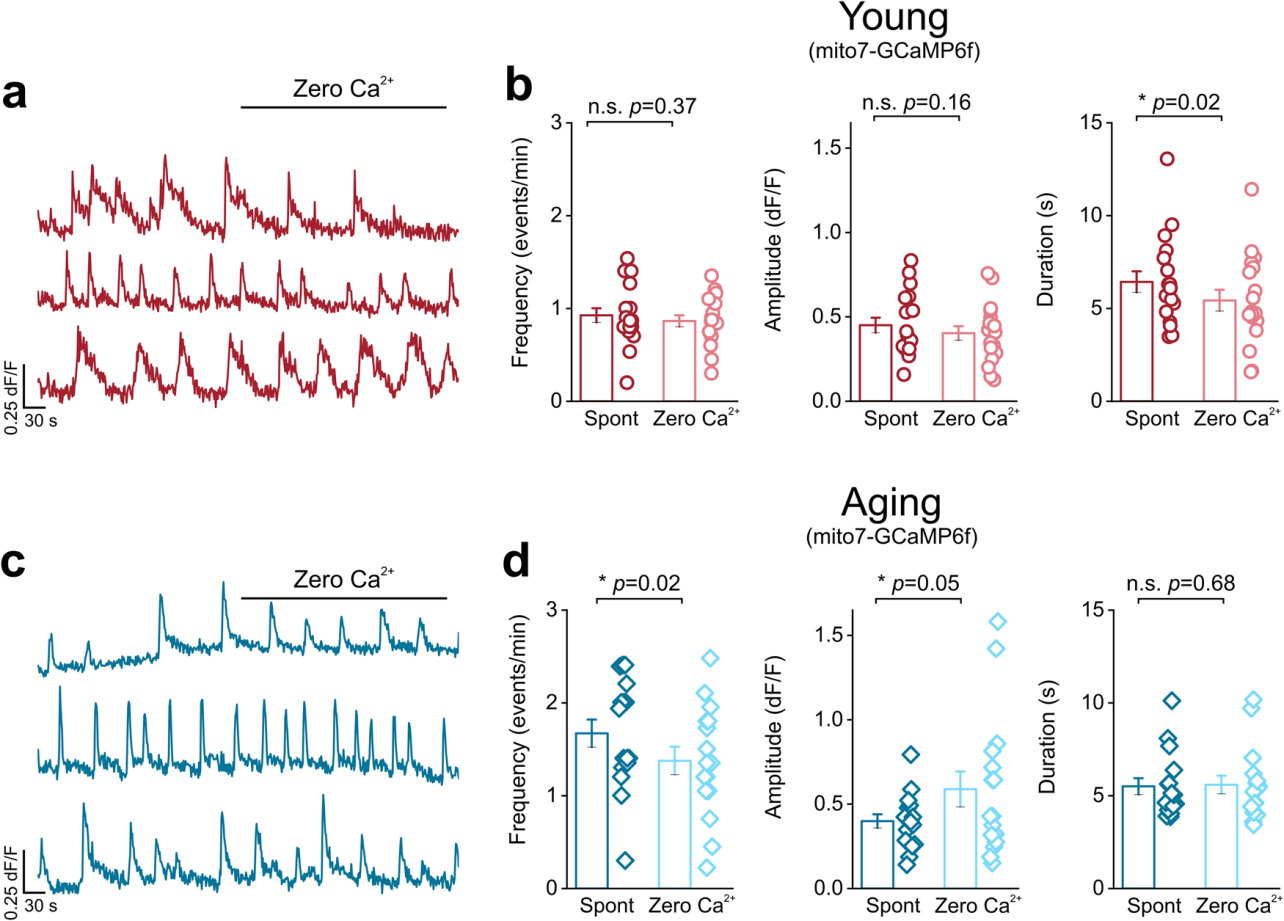
Endfoot mitochondria in young and aging mice do not rely on extracellular Ca^2+^. Representative mitochondrial Ca^2+^ event traces from young (**a**) or aging (**c**) mice before and after bath application of zero Ca^2+^ aCSF. ROIs were averaged across individual blood vessels to compare frequency (*left*), amplitude (*middle*), and duration (*right*) for mitochondrial Ca^2+^ events recorded in young (**b**) or aging (**d**) mice. For young mice, *n* = 52 ROIs and 18 blood vessels from 3 mice. For aging mice, *n* = 28 ROIs and 16 blood vessels from 5 mice. Error bars are S.E.M. All frequency and duration *p* values and the aging amplitude *p* value are based paired sample t-tests and the young amplitude *p* value is based on a Wilcoxon signed ranked test.

**Fig. 9:**
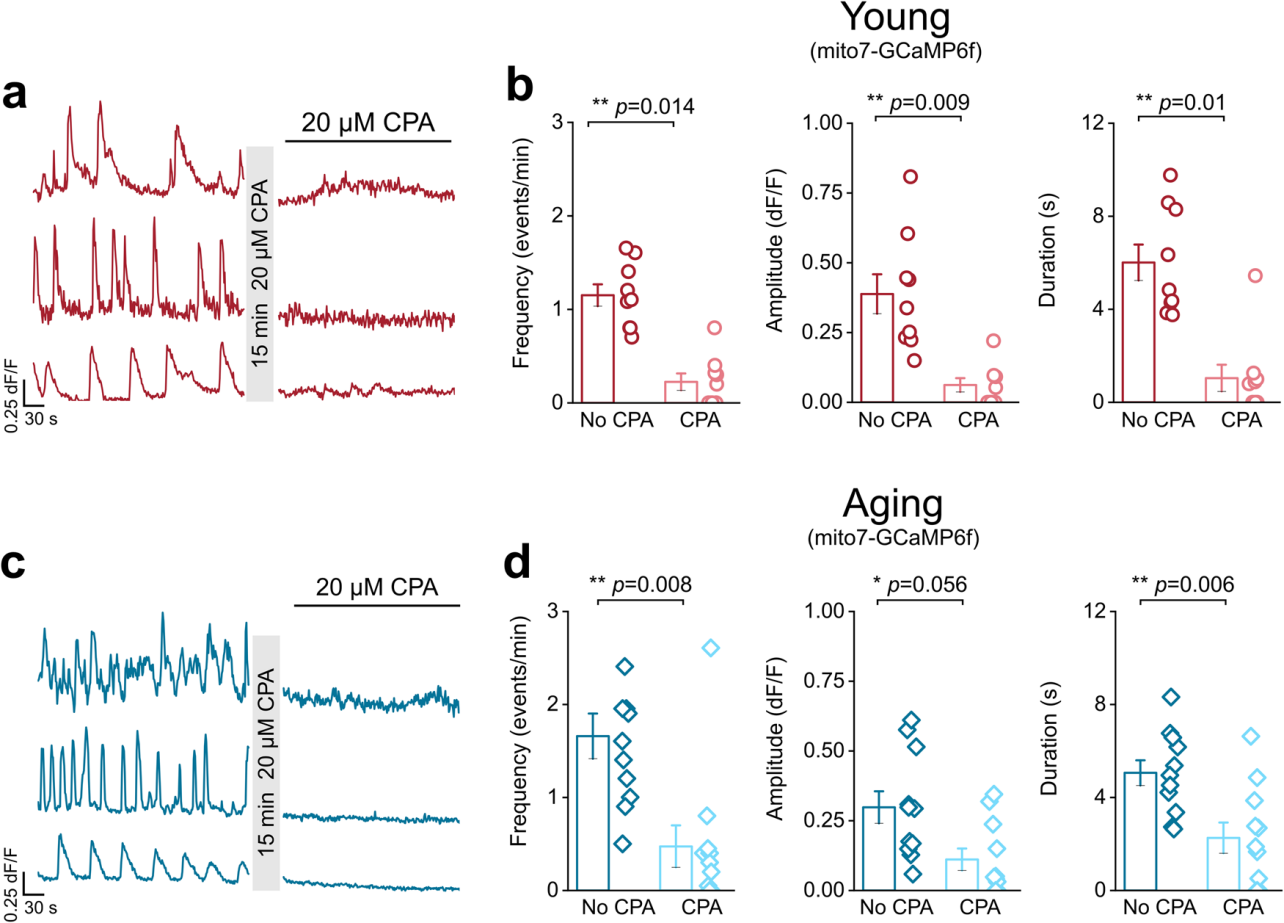
Endfoot mitochondria in young and aging mice depend on intracellular Ca^2+^ stores. Representative mitochondrial Ca^2+^ event traces from young (**a**) or aging (**c**) mice before and after bath application of CPA. (**b** and **d**) ROIs were averaged across individual blood vessels to compare frequency (*left*), amplitude (*middle*), and duration (*right*) for mitochondrial Ca^2+^ events recorded in young (**b**) or aging (**d**) mice. For young mice: *n* = 22 ROIs and 9 blood vessels from 3 mice and for aging mice: *n* = 18 ROIs and 11 blood vessels from 5 mice All errors are SEM. All frequency, amplitude, and young duration *p* values are based on Wilcoxon signed ranked tests. The aging duration *p* value is based on a paired sample t-test.

Due to the observed increase in reliance on ER Ca^2+^ stores within aging astrocyte endfeet, and a significant aging-induced increase in the frequency of Ca^2+^ influx into endfoot mitochondria, we asked if ER Ca^2+^ stores are a primary source for Ca^2+^ influx into astrocytic endfoot mitochondria for both young and aging mice. To test this idea, we sequentially depleted extracellular Ca^2+^ with zero Ca^2+^ aCSF, followed by depletion of intracellular ER Ca^2+^ stores with 20 µM CPA in DLS slices. In each case, we measured the kinetics of Ca^2+^ influx into endfoot mitochondria within the DLS of young and aging mice. Extracellular Ca^2+^ depletion with zero Ca^2+^ aCSF did not alter the frequency, amplitude, or duration in young mice and caused only modest changes in endfoot mitochondrial Ca^2+^ influx in aging mice (Fig. 8; Supplemental movies 8 and 9). In contrast, depletion of ER Ca^2+^ stores with CPA resulted in a significant ~80% decrease in the frequency, amplitude, and duration of mitochondrial endfoot Ca^2+^ influx in young mice (Fig. 9b; Supplemental movie 10). In aging mice, we observed a significant 72% decrease in frequency, 63% decrease in amplitude, and 55% decrease in event duration (Fig. 9d; Supplemental movie 11). These data confirm that the ER is indeed a primary source for Ca^2+^ influx into astrocytic endfoot mitochondria for both young and aging mice.

## Discussion

It is well established that neurovascular impairment can initiate neurodegeneration and that aging is associated with changes in astrocytic Ca^2+^ homeostasis^36,42,43,5–12^. Though seemingly unrelated, these findings suggest that aging could alter Ca^2+^-mediated coupling of astrocyte endfeet and cerebral vasculature; thereby, leading to neurodegeneration. Here, we generated a protocol in acute mouse brain slices to measure spontaneous astrocyte endfoot Ca^2+^ signals adjacent to fluorescently labeled striatal blood vessels with subcellular resolution at multiple timepoints across the lifespan. The findings of this study identify alterations to spontaneous Ca^2+^ events in astrocyte endfeet of aging mice that are potentially pathological.

In this study, we quantified three parameters of Ca^2+^ signals, frequency, amplitude, and duration. These parameters respectively determine the number of events occurring, the relative amount of Ca^2+^ fluxing between cellular compartments, and the length of time Ca^2+^ is mobilized. We found that aging caused spontaneous endfoot Ca^2+^ events to become larger, longer-lasting, and propagate faster than those in young or middle-aged mice (Figs 1 and 2). Previously, Ronco et al. observed that hippocampal astrocytes isolated from 3xTg-AD mice exhibited abnormally large Ca^2+^ signals after treatment with amyloid beta oligomers; indicating that dysregulated Ca^2+^ mobilization is associated with hallmarks of neurodegenerative disease^44^. However, whether changes in astrocyte endfoot Ca^2+^ mobilization specifically contribute to neurovascular uncoupling remain to be explored. Additionally, the aging-induced effects on endfoot Ca^2+^ signals we report are likely localized to the endfoot compartment, as a study by Gómez-Gonzalo et al. showed that Ca^2+^ events in astrocyte soma are similarly responsive to neurotransmitter release in mice aged 5–20 months^45^.

We found that aging astrocyte endfeet in the DLS demonstrate a significant reduction in CALR (Fig. 5). ER-localized CALR plays critical roles in Ca^2+^ buffering, quality control for protein folding, and ER-mitochondria Ca^2+^ transfer^46–48^. Multiple reports have demonstrated that reduced CALR expression in aging neurons and glial cells begins in middle-age (12 months) and is associated with cognitive decline^49,50^, decreased ATP production^51^, changes to mitochondrial morphology^51^, and increased accumulation of misfolded proteins^52^. Moreover, a study by Biwer et al. demonstrated that conditional deletion of CALR from endothelial cells in aging mice increased the spatial spread of spontaneous endothelial Ca^2+^ signals and impaired Ca^2+^ mobilization^47^. Thus, we hypothesize that our observation of decreased CALR expression is upstream of the changes to spontaneous Ca^2+^ events in the cytosol and mitochondria of DLS astrocyte endfeet reported in Figs 1, 2, 4, and 6. As such, future studies to determine the extent to which decreased endfoot CALR expression and how its downstream effects potentiate neurodegeneration or BBB impairment are warranted.

Our finding that endfoot Ca^2+^ signals in aging mice rely heavily on ER Ca^2+^ stores (Fig. 4d) strongly suggests that the observed changes in membrane-associated Ca^2+^ signal kinetics reflect Ca^2+^ efflux from the ER into the cytosol of astrocytic endfeet. In this regard, our finding that endfoot Ca^2+^ signal frequency in aging mice does not decrease with exposure to zero calcium aCSF (Fig. 3d) implies minimal contribution of ion channels such as TRPA1 in astrocytes. Studies show that TRPA1 is expressed in the aging mouse brain^53^ and databases of the aging human brain clearly demonstrate TRPA1 expression^54–56^. In the context of these findings, it is important to note that the zero calcium aCSF used in our experiments (Figs 3, 8) would contain residual Ca^2+^, which is sufficient to result in Ca^2+^ signals via astrocytic ion channels. Despite this technical limitation, we observed a significant decrease in Ca^2+^ signals from young and middle-aged astrocyte endfeet.

The ER in astrocyte endfeet are morphologically and functionally coupled with mitochondria^34,57^, enabling mitochondria to sequester Ca^2+^ released from the ER. We found a significant decrease in the number of astrocyte endfoot mitochondria in the aging DLS (Fig. 6). Similarly, a study in the aging cerebral cortex showed a significant decrease in BV mitochondria^58^; however, BV mitochondria in the vascular-rich striatum have yet to be quantified. Conversely, a prior study in the aging striatum demonstrated an increase in mitochondrial fragmentation for neuronal processes, indicating an increase in mitochondrial mass^59^. Taken together, these findings demonstrate that changes in mitochondrial content are heterogeneous and likely depend not only on the type of brain cell being studied but also depend on the specific brain region that is being considered. Another downstream target of aging-related alterations in Ca^2+^ efflux from the ER of astrocytic endfeet are changes to mitochondrial Ca^2+^ signals. In line with this rationale, we showed aging-related increases in Ca^2+^ efflux from the ER were associated with increases to the frequency of Ca^2+^ influx events of endfoot mitochondria (Fig. 6d). Compromised endfoot mitochondrial function could have negative consequences on endfoot integrity and neurovascular function. In support of this idea, a recent study has shown that astrocyte-specific conditional deletion of mitofusin-2, an outer mitochondrial membrane GTPase involved in tethering mitochondria to ER results in increased fragmentation of endfoot mitochondria, alterations in mitochondrial Ca^2+^ flux frequency, and a significant loss of vascular repair after injury^34^.

Reduced CALR expression has been linked to the loss of motoneurons during amyotrophic lateral sclerosis (ALS)^60^, and physical and functional changes in the NVU have been established as an important contributor to neurodegenerative disorders such as AD and PD^5–12^. Thus, one downstream consequence of reduced CALR expression and altered Ca^2+^ signals in aging astrocytic endfeet may be the gradual loss of AQP4, which could lead to BBB impairment given the importance of normal AQP4 localization and function in maintaining BBB integrity^61^. Here, we report an age-related decrease in AQP4 expression for DLS astrocyte endfeet but surprisingly no difference in DLS BV density (Fig. 6). In line with our finding are studies showing no change in striatal BV density between young and aged mice^62,63^. However, numerous reports confirm decreased cortical BV density with aging^64–66^; indicating that BV density loss in the aging brain is likely region specific.

In summary, we show that aging-related abnormalities in spontaneous astrocyte endfoot cytosolic and mitochondrial Ca^2+^ events are downstream of reduced CALR expression. This study does not address whether changes in Ca^2+^ signals and CALR/AQP4 expression in the aging endfoot are directly associated with BBB dysfunction. In particular, studies characterizing the relationship between ER stress, CALR expression, and changes to vascular permeability are justified. In support of this idea, reports show that ER stress is associated with vascular endothelial cell dysfunction^67,68^ and angiogenesis in the context of disease^69^. Therefore, because reduced CALR can increase ER stress^60^, it is possible that CALR-induced alterations in ER stress within the aging astrocyte endfoot can affect BBB integrity. Future work will focus on dissecting the relationship between CALR in astrocytic endfeet, ER stress, and BBB permeability.

## Materials and methods

### Mice

Male and female C57BL/6J mice (Jackson laboratory, Bar Harbor, ME, USA) were used for all experiments. Young mice were 3–4 months of age, middle-aged mice were 12-15 months of age, and aging mice were 20–30 months of age. Mice were aged in-house in the animal vivarium. All experiments were conducted in compliance with all relevant ethical regulations for animal testing and research approved by the Texas A&M University Institutional Animal Care and Use Committee. Food and water were provided ad libitum. All mice were maintained on a 12 h light-dark cycle.

### Adeno-associated virus (AAV) injection into the DLS of mice

Young, middle-aged, and aging mice were deeply anesthetized using isoflurane dispensed from a SomnoSuite Low Flow Anesthesia System (Kent Scientific, Torrington, CT), and a craniotomy was performed as previously described^41,70^. AAVs were injected into the right DLS using a glass injection pipette, at a rate of 750 nl/min using a Harvard Apparatus Pump 11 Pico Plus Elite, 70-41506 (Harvard Apparatus, Holliston, MA). To image near membrane astrocyte endfoot Ca^2+^ events in young, middle-aged, and aging mice, 1 μl of AAV2/5-GfaABC1D-Lck-GCaMP6f (10^13^ genome copies/ml) (Addgene viral prep # 52924-AAV5) was injected into the DLS. In young, middle aged, and aging mice, astrocyte endfoot mitochondrial Ca^2+^ events were imaged by injecting 2 μl of AAV2/5-GfaABC1D-mito7-GCaMP6f (10^13^ genome copies/ml)^41^ into the DLS. Coordinates for stereotaxic injection were 0.8 mm rostral to bregma, 2 mm lateral to the midline and 2.4 mm ventral to the pial surface.

### Confocal imaging of acute brain slices

Young, middle-aged, and aging mice were deeply anesthetized with isoflurane and the left ventricle was rapidly accessed. 200 μl of DyLight 594 labeled *Lycopersicon esculentum* lectin (tomato lectin, TL; DL-1177, Vector Laboratories, Burlingame, CA) was injected into the apex of the beating left ventricle. 1 min following TL injection into the left ventricle, the mouse was decapitated, the brain was extracted and then immediately blocked for obtaining live brain slices. 400 µm coronal striatal slices were cut using a Microslicer 01 N (Ted Pella) in a solution comprising 194 mM sucrose, 30 mM NaCl, 4.5 mM KCl, 1.2 mM NaH_2_PO_4_, 26 mM NaHCO_3_, 10 mM D-glucose, and 1 mM MgCl_2_ saturated with 95% O_2_ and 5% CO_2_. Live brain slices were incubated in artificial cerebrospinal fluid (aCSF) composed of 126 mM NaCl, 2.5 mM KCl, 1.24 mM NaH_2_PO_4_, 1.3 mM MgCl_2_, 2.4 mM CaCl_2_, 26 mM NaHCO_3_, 10 mM D-glucose at 33 °C for 30 min and then maintained at 23 °C in aCSF for the duration of the experiment.

Live mouse striatal brain slices were imaged with an Olympus FV1200 upright laser-scanning confocal microscope using a 40× water immersion objective lens (NA 0.8), and a digital zoom of 3. We used a 488 nm excitation wavelength at 10% of maximum intensity of a 100 mW Argon laser to record membrane-associated astrocyte endfoot and mitochondrial Ca^2+^ events. A 25 mW HeNe 594 nm excitation laser line at 10% of maximum intensity was used to visualize blood vessels (BVs) and localize astrocytic endfoot Ca^2+^ events in the DLS. Membrane-associated endfoot and mitochondrial Ca^2+^ events near TL-labeled BVs were identified as astrocytic endfoot Ca^2+^ events and imaged for 10 min at 1 frame per second. Imaging parameters (laser intensity, HV, gain, offset, aperture diameter) were maintained constant across all imaging sessions.

Imaging of Ca^2+^ events in astrocyte endfeet was performed by first imaging spontaneous Ca^2+^ events, followed by sequential depletion of extracellular Ca^2+^ and ER Ca^2+^ in the slice. To deplete extracellular Ca^2+^, spontaneous Ca^2+^ events were recorded for 5 min in bath perfused aCSF, followed by bath perfusion of zero Ca^2+^ aCSF, in which CaCl_2_ was omitted. To deplete ER Ca^2+^ stores, 20 µM cyclopiazonic acid (CPA, Abcam, Cambridge, MA, ab120300) was perfused into the bath for 15 min, after which Ca^2+^ events were recorded for 5 min from the same field of view.

### Immunohistochemistry

Mice were deeply anesthetized with isoflurane and transcardially perfused with 1X PBS, followed by 10% formalin. Brains were extracted and stored in 10% formalin for 48 h at 4 °C then dehydrated in 30% sucrose in 1X PBS (Sigma, St Louis, MO, cat# S7903) for 48 h. A Microm HM 550 cryostat was used to cut 40 µm coronal sections of the striatum that were stored at 4 °C in 0.01% sodium azide (Sigma, cat# S2002) until the day of immunostaining. Sections were washed 3× in 1X PBS, then permeabilized and blocked in 0.5% Triton X-100 and 10% normal goat serum at room temperature for 45 min. To prevent cross reactivity, sections were sequentially stained with calreticulin (CALR) and aquaporin 4 (AQP4) primary and secondary antibodies. Sections were first incubated overnight at 4 °C in rabbit anti-CALR (1:500, ThermoFisher, Waltham, MA, cat# PA3900), washed twice in 1X PBS, then incubated in goat anti-rabbit Alexa Fluor 594 (1:2000, Abcam, cat# ab150077) secondary antibody for 1 h at room temperature. After 3 washes in 1X PBS, sections were stained with rabbit anti-AQP4 1:5000 (Alomone, cat# AQP-004) overnight at 4 °C, followed by goat anti-rabbit Alexa Fluor 488 (1:2000, Abcam, cat# ab150176) for 1 h at room temperature. For astrocyte mitochondria images, the right DLS of young and aging mice was injected with 400 nl of AAV2/5-GfaABC1D-mito7-eGFP (10^13^ genome copies/ml), three weeks later mice were perfused, and brains from young and aging mice were collected. 40 µm striatal sections were obtained and then stained with rabbit anti-AQP4 and goat anti-rabbit Alexa Fluor 595. Following all staining, sections were mounted on glass slides and imaged using an FV1200 inverted confocal microscope equipped with a 60× oil immersion objective. Settings for laser power, high voltage (HV), gain, offset, and aperture diameter were maintained across all sessions.

For CALR images in the DLS and CTX, single plane images were aquired at 60× and 3× digital zoom using AQP4 staining to locate BVs. To analyze CALR intensity, ROIs were generated for the whole FOV using the rectangle tool or manually demarcated around AQP4 labeled BVs with the polygon tool using ImageJ. To count the number of CALR particles in an AQP4 labeled BV, images were manually thresholded and the Analyze Particles plugin on ImageJ was used with an ROI contraint of 0.01–1000 µm^2^. To quanitfy the number of DLS endfoot mitochondria, single plane images were aquired at 60× and 3× digital zoom using AQP4 staining to locate BVs. AQP4 ROIs were manually generated with the polygon tool in ImageJ, thresholded, and analyzed with the Analyze Particles plugin. AQP4 intensity was also quantified using the same AQP4 ROI. For AQP4 BV density imaging, z-stacks with a 1.30 µm step size were acquired at 20×. A z-projection was generated for each DLS image and AQP4 labeled BVs were manually counted.

### Analysis of Ca^2+^ events in astrocytic endfeet

Membrane-associated and mitochondrial astrocytic endfoot Ca^2+^ events were identified as Ca^2+^ events occurring immediately adjacent to the abluminal side of TL-labeled DLS BVs. Regions of interest (ROIs) for endfoot Ca^2+^ events were generated as previously described, using the ImageJ plugin, GECIquant^71^. dF/F traces of GCaMP6f fluorescent signals were generated from the acquired ROIs and analyzed with Minianlysis 6.0.07 (Synaptosoft) to generate amplitude (dF/F), and duration (s) values for each trace. Frequency (events/min) was determined by manually counting the number of peaks associated with Ca^2+^ events that initiated at a specific ROI. Expanding Ca^2+^ waves coming from adjacent ROIs were visually identified and omitted from the ROI being used for determining event frequency.

For expanding endfoot Ca^2+^ waves, the velocity (µm/s), distance traveled (µm), and area (µm^2^) of membrane-associated astrocytic endfoot Ca^2+^ events were analyzed using ImageJ. All confocal movies for calculating expansion velocity were acquired at a frame rate of 1 frame/s. Expansion velocities were determined by counting the number of frames taken for Ca^2+^ events to attain maximum area within the astrocytic endfoot. The distance covered by expanding endfoot Ca^2+^ signals was determined by using the line tool on ImageJ to trace the maximum length attained by Ca^2+^ events along the corresponding BV. The area of propagating Ca^2+^ events were quantified using the polygon tool in ImageJ. Using the polygon tool, the total area covered by a propagating Ca^2+^ event along the BV was manually outlined from the final frame of the Ca^2+^ event. In the case of ROIs with multiple areas, the areas were averaged.

AQuA^72^ was used to determine directionality of expanding Ca^2+^ events. Prior to analysis videos were cropped to specifically encompass BVs, and BVs were rotated to the horizontal orientation. Videos were then loaded into AQuA using the default GCaMP-ex vivo-lck preset with a nominal spatial resolution of 0.21 µm/pixel and a temporal resolution of 1 frame per second. Two ROIs were then manually drawn to demarcate the area to be analyzed and to identify the BV as a landmark. The top of the FOV was assigned as north and this direction was maintained for each video. All parameters during the detection pipeline were adjusted to minimize the detection of noise for each video. Specific values for each parameter did not differ more than 5% from the software presets. During post processing, detected events smaller than 10 µm^2^ or that occurred more than 5 µm away from the BV ROI were excluded. An overall propagation score, defined as an arithmetic sum of 4 directions for each endfoot calcium event was generated using AQuA. Only events with an overall propagation score greater than 1 were determined to be expanding, while any event with a propagation score less than 1 was considered a static event and was excluded. For expanding events, we utilized a ratio of posterior to anterior propagation score as a method to determine overall directionality. For example, an event with a posterior/anterior value greater than 1 was determined to be moving towards the BV.

### Sampling and statistics

In all graphs each datapoint represents a single DLS BV whereby all ROIs pertaining to that BV have been averaged. Statistics were performed using Origin 2022 (OriginLab, Northampton, MA). For statistical testing, datasets were first tested for normality. Non-normal datasets were subjected to either a Mann-Whitney or paired sample Wilcoxon signed rank test. A two-sample *t* test or paired sample *t* test was used to compare normally distributed datasets. *p* < 0.05 was considered statistically significant. Sample sizes, statistical tests used, and exact *p* values are reported in figures and figure legends.

### Reporting summary

Further information on research design is available in the Nature Research Reporting Summary linked to this article.

## Supplementary information

Reporting Summary

Supplemental Movie 1

Supplemental Movie 2

Supplemental Movie 3

Supplemental Movie 4

Supplemental Movie 5

Supplemental Movie 6

Supplemental Movie 7

Supplemental Movie 8

Supplemental Movie 9

Supplemental Movie 10

Supplemental Movie 11

## Data Availability

The datasets generated and/or analyzed for this study are available from the corresponding author upon reasonable request.
